# Comorbid Symptoms Occurring During Acute Low-Tone Hearing Loss (AHLH) as Potential Predictors of Menière's Disease

**DOI:** 10.3389/fneur.2018.00884

**Published:** 2018-10-29

**Authors:** Katharina Stölzel, Judith Droste, Linda Josephine Voß, Heidi Olze, Agnieszka J. Szczepek

**Affiliations:** ^1^Department of Otorhinolaryngology, Head and Neck Surgery, Charité- Universitätsmedizin Berlin, Corporate Member of Freie Universität, Berlin Humboldt Universität zu Berlin and Berlin Institute of Health, Berlin, Germany; ^2^Department of Audiology and Phoniatrics, Charité- Universitätsmedizin Berlin, Corporate Member of Freie Universität, Berlin Humboldt Universität zu Berlin and Berlin Institute of Health, Berlin, Germany

**Keywords:** acute low-frequency hearing loss, early diagnosis of Menière's disease, sole symptom, comorbid tinnitus, pure tone audiometry

## Abstract

Acute low-tone sensorineural hearing loss (ALHL) is a type of idiopathic sudden sensorineural hearing loss. ALHL is rarely a solitary condition but rather co-occurs with vertigo and tinnitus, being an element of contemporary diagnostic criteria for Menière's disease (MD). The goal of our present study was to determine the value of ALHL for the early diagnosis of MD in patients presenting in the emergency room with ALHL as a main complaint. The files of 106 patients with ALHL who were admitted to the emergency room over the period of 7 years and 104 patients with acute high- tone sensorineural hearing loss (AHHL) from the same period were included in this retrospective study. Forty ALHL patients presented with recurrent episode of hearing loss and 66 remaining patients presented with ALHL for the first time. Of the latter group, 25 patients gave consent for the follow-up. First, we analyzed the difference in the occurrence of tinnitus and vertigo between the ALHL and AHHL groups. In patients with ALHL, the incidence of vertigo with tinnitus and the number of recurrent episodes were statistically higher than in patients with AHHL. Next, we focused on the ALHL follow-up group (25 patients). In that group, two patients had all MD symptoms at presentation, 18 had ALHL and tinnitus and five ALHL only. Of 18 patients with ALHL and tinnitus at admission, five developed vertigo and thus the triad of Menière's disease. None of the five patients with AHLH as a sole symptom developed MD during the follow-up time but four of them have developed tinnitus. Patients with recurrent ALHL had significantly higher incidence of MD than the patients with first episode. We conclude that some patients who present with ALHL and concomitant tinnitus or have recurrent episodes of ALHL are more likely to develop Menière's disease than these patients, who present with ALHL as a sole symptom. Nonetheless, we recommend otological follow-up for all patients presenting with ALHL.

## Introduction

Reported incidence of sudden idiopathic sensorineural hearing loss (SSNHL) varies between the countries and ranges between 27/100,000 in the United States (1) and 168/100,000 in Germany (2). Based on the frequencies affected by the hearing loss, SSNHL can be divided into two subgroups: the acute high-frequency hearing loss (AHHL), consisting of roughly 80% of SSNHL cases and an acute low-frequency sensorineural hearing loss traditionally called *acute low-tone hearing loss* (ALHL), consisting of 17 to 23% of all SSHL cases (3). The low incidence of ALHL is the reason behind infrequent studies and rather small number of cases included in most of the ALHL reports (4–7). In one large Japanese study, 642 patients diagnosed with ALHL were evaluated over a period of 24 years in a national study involving 23 hospitals (8). Another multi-center study conducted at 30 hospitals from April 2014 to March 2016 reported 931 cases of ALHL (9). However, the diagnostic significance of ALHL as a main complaint for the Meniere's disease (MD) reminds open.

Diagnostic criteria for MD include attacks of vertigo, fluctuating ALHL, tinnitus and fullness in ear. Despite intensive research, the etiology of MD is still under debate. In addition, the diagnosis of MD remains challenging and subject of clinical and scientific discussions in contemporary literature. In the year 2015, Barany Society came up with new diagnostic criteria for MD that differentiate between *definite MD* and a *probable MD*. In addition to two or more episodes of vertigo and fluctuating aural symptoms such as tinnitus or ear fullness, the diagnostic criteria of definite MD include low- up to middle-tone unilateral hearing loss during or after the vertigo attack documented by pure tone audiometry. Hearing loss in the affected ear should be not less than 30 dB in at least two frequencies below 2,000 Hz, as compared to the contralateral ear. If both ears are affected, then the hearing loss should be at least 35 dB, affecting at least two frequencies below 2,000 Hz (10).

Hearing loss can occasionally precede the vertigo attacks by months or even years, consistent with so called delayed Menière's Disease, therefore hindering the definite diagnosis (11). Because the onset of MD typically coincides with hearing loss, the interpretation of pure tone audiogram is a subject of intense research. Morita et al. suggested that ALHL is defined by the sum of hearing thresholds ≥ 70 dB in the frequencies 0.125, 0.250, and 0.5 kHz and the sum of the hearing thresholds ≤ 60 dB in the frequencies 2, 4 and 8 kHz (12). Further studies have shown that MD patients with ALHL, frequently had endolymphatic hydrops (13, 14). Junicho et al. found that patients with recurrent ALHLs are more likely to be affected by endolymphatic hydrops than patients with single ALHL episode. In that particular study, 10% of patients developed *definite MD* (15).

In our study, we wanted to assess the significance of ALHL reported by the patients as a sole or main complaint, for the prognosis of MD. Therefore, we characterized a group of patients with ALHL using as a control a group of patients with acute high-tone sensorineural hearing loss (AHHL).

## Patients and methods

**Figure 1:**
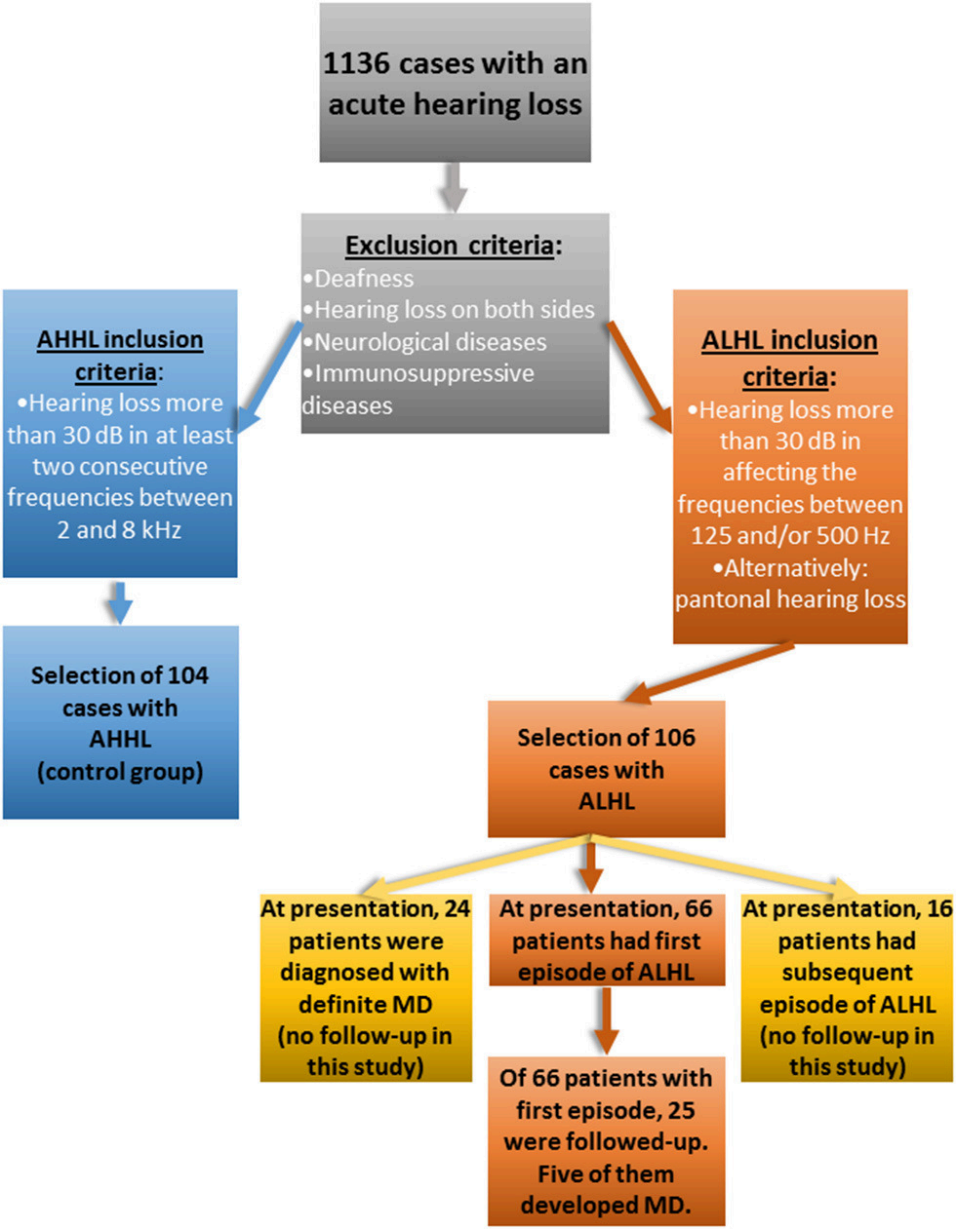
Flow chart of the study.

Local Ethics Committee granted a permit for this retrospective study (permit number EA1/116/14). First, we analyzed the data of 1,136 patients who were admitted to the ER in our hospital over the period of 7 years (2007–2013) with a sudden idiopathic sensorineural hearing loss (SSNHL) that occurred no longer than 3 weeks prior to admission. One hundred and six patients with ALHL, who met the criteria of pure tone audiometry characteristic for MD, were included in the study. As a control group, 104 patients with an acute high- tone hearing loss (AHHL) were included. All of these patients (ALHL and AHHL) were seeking medical help solely because of sudden hearing loss (see Figure 1) and some of them had additional symptoms, such as tinnitus or vertigo; which, however, were not the reason for their hospital admission.

Exclusion criteria included deafness, bilateral hearing loss, neurological diseases and immunological diseases (Figure 1). The presence of additional symptoms such as tinnitus and vertigo was recorded as an answer to closed question “do you have tinnitus?” or “do you have vertigo?.” Tinnitus-oriented diagnosis was not performed, as ER in our hospital is not equipped for this type of medical investigations. In addition, number of episodes prior to admission (no episodes vs. recurrent episodes) was recorded and analyzed.

In the ALHL group of patients, the first episode of hearing loss was determined for 66 patients, 24 patients had the ALHL for the second time, 5 patients had the third episode and 11 patients had more than three episodes of ALHL. Of 40 patients with more than one episode of ALHL, 16 reported having vertigo.

### Statistical analyses

The statistical description and evaluation were performed using the IBM SPSS Statistics software version 23. For the analyses of frequency differences between the variables, we used the chi-squared test with the alpha value <0.05. Additionally, we used sensitivity/ specificity/ positive predictive value/ negative predictive value of ALHL to diagnose Meniere's disease in comparison to the control group. The predictive value was computed using Screening Validity feature of SPSS. Positive predictive value for the presence of individual symptoms is the probability that subjects who have given symptom will develop MD. Negative predictive value is the probability that subjects who do not have given symptom will not have the disease.

## Results

### Age and gender

Of 106 patients with ALHL, 63 (59.4%) were men and 43 (40.6%) women, whereas in the control group of 104 patients with AHHL, 71 (68.3%) were men and 33 (31.7%) were women. The analysis indicated no statistically significant differences in gender representation between the groups.

The average age of the ALHL patients was 45.01 ± 13.86 within a range 18–75 years whereas the age of control group (AHHL) was on average 48.49 ± 14.35 years (range 20–80). There were no significant differences in age between the groups. However, there was a significant difference when the age of patients at the occurrence of first episode was compared, suggesting that ALHL patients were significantly older at first presentation (mean age 47.92) than the AHHL patients age at first presentation (mean age 44.55; *p* = 0.0001). Despite its statistical significance, this finding is of minute clinical relevance.

### Comparison between ALHL and AHHL groups regarding comorbid symptoms

1. Tinnitus as comorbid symptom
  There were no significant differences in the numbers of comorbid tinnitus cases between the two sudden hearing loss groups (Table 1, chi-squared test, *p* = 0.145).
2. Vertigo as comorbid symptom
  The number of comorbid vertigo cases was significantly higher in ALHL group (Table 2, chi-squared test, *p* = 0.0001). In the ALHL group, 31 patients reported having vertigo.
3. Tinnitus and vertigo as comorbid symptoms (Triad of MD)
  The number of comorbid vertigo *and* tinnitus cases was significantly higher in ALHL group (Table 3, chi-squared test, *p* = 0.002). The triad of MD occurred in 24.5 % of ALHL group.
4. Frequency of repeated episode as comorbid symptom
  The incidence of repeated hearing loss episodes was significantly higher in ALHL group (Table 4, chi-squared test, *p* = 0.002).

**Table 1:** Tinnitus as comorbid symptom of ALHL.

		**Tinnitus**		**Sum**
		**positive**	**negative**	
Type of SSNHL	ALHL	80	26	106
	AHHL	69	35	104
	Sum	149	61	210

Sensitivity: 54.0 %, Positive predictive value: 75.5 %.

*Specificity: 57.4 %, Negative predictive value: 33.7 %*.

**Table 2:** Vertigo as comorbid symptom of ALHL.

		**Vertigo**		**Sum**
		**positive**	**negative**	
Type of SSNHL	ALHL	31	75	106
	AHHL	9	95	104
	Sum	40	170	210

Sensitivity: 77.5 %, Positive predictive value: 29.2 %.

*Specificity: 55.8 %, Negative predictive value: 91.3 %*.

**Table 3:** Tinnitus and vertigo as comorbid symptoms of ALHL.

		**Tinnitus and vertigo**		**Sum**
		**positive**	**negative**	
Type of SSNHL	ALHL	26	80	106
	AHHL	8	96	104
	Sum	34	176	210

Sensitivity: 76.5 %, Positive predictive value: 24.5 %.

*Specificity: 54.5 %, Negative predictive value: 92.3 %*.

**Table 4:** Frequency of recurrent SSNHL as comorbid symptom.

		**Repeated episode**		**Sum**
		**positive**	**negative**	
Type of SSNHL	ALHL	40	66	106
	AHHL	19	85	104
	Sum	59	151	210

Sensitivity: 67.8 %, Positive predictive value: 37.7 %.

*Specificity: 56.3 %, Negative predictive value: 81.7 %*.

### Differences within the ALHL group

**Figure 2:**
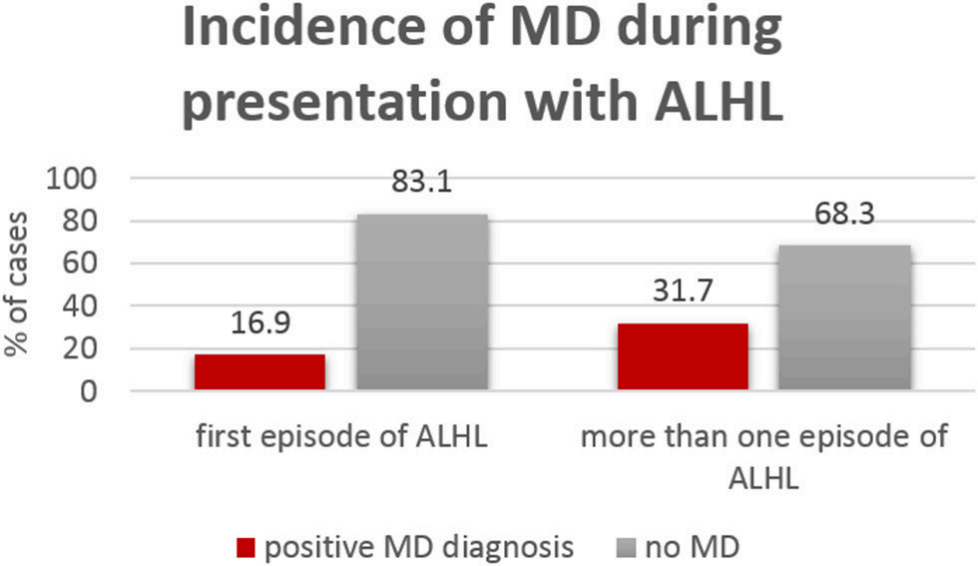
Incidence of definite MD within the group presenting for the first time with ALHL (65 patients) and the group presenting with recurrent ALHL (41 patients).

In the group of patients with recurrent ALHL episodes, the incidence of MD (Figure 2) was twice as high (37.1%) as in the group with the first episode of hearing loss (16.7%).

Full diagnostic triad characteristic of MD was diagnosed at admission in 9 patients with first ALHL episode, 9 patients with the second ALHL episode, one patient with third ALHL episode and in 5 patients with more than 3 ALHL episodes (total 24 patients).

**Figure 3:**
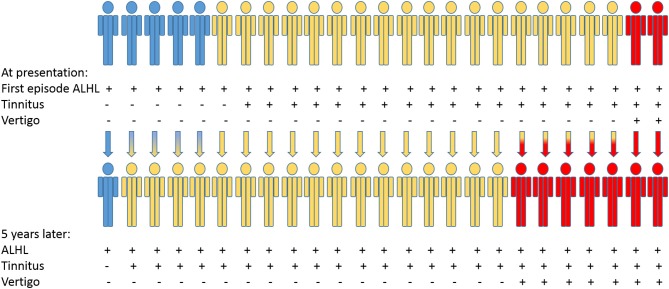
Flowchart visualizes changes in the MD-related health status of 25 patients with first episode of ALHL as a main complaint, who were followed up for 5 years.

Of 66 patients who had the ALHL for the first time, 25 patients were followed for 5 years upon signing a written consent: five presented with sole ALHL, 18 presented with ALHL and tinnitus and two had full triad at presentation. Of five patients who presented with sole ALHL, four had developed tinnitus but reported no other symptoms during the follow up time. Of 18 patients who at presentation reported tinnitus in addition to ALHL, five developed vertigo during the follow up; thus, qualified for the diagnosis of *definite MD* (Figure 3).

The results of pure tone audiometry performed during the admission were analyzed in subgroups of ALHL—“ALHL right ear affected” and “ALHL-left ear affected”. The scores for each of the frequencies tested (250, 500, 1,000, 1,500, 2,000, 3,000, 4,000, 6,000, and 8,000 Hz) were compared individually between MD and non-MD patients using one-way ANOVA and taking under account the affected side. We found no statistical differences in pure tone audiograms between the MD and non-MD groups.

There were more patients with greater hearing loss in the “full MD symptoms” group than in the “incomplete symptoms” group. In detail, of 24 patients who had full triad of MD symptoms, 10 patients (42% of that group) had hearing loss between 30 and 60 dB and 14 (58%) had a hearing loss of more than 60 dB. Of 82 ALHL patients without full triad of MD symptoms, 52 (63% of that group) had a hearing loss between 30 and 60 dB and 30 (37%) had a hearing loss more than 60dB.

### Comorbid conditions within ALHL group

In recent years, MD has been classified into five subtypes. Of them, type 4 MD is characterized by comorbid migraine (16–18) whereas type 5 by comorbid autoimmune disorders (19). To determine the prevalence of this two types of comorbidities in our cohort of subjects with ALHL, we searched for them in the medical history which was available. We found no mention of migraine headache. However, five of 106 patients with ALHL had autoimmune diseases. In detail, three patients had Hashimoto disease, one patient had Sjögren syndrome as well as lupus and one patient had type 1 diabetes. Of the five autoimmune-positive patients, two belonged to the group with definite MD at the presentation with ALHL and three belonged to the group who presented with ALHL for the first time but did not give their consent for further follow-up.

## Discussion

Low-tone hearing loss is one of the diagnostic parameters used to identify definite Menière's disease (10). Although infrequent, the sudden low-tone hearing loss ALHL occurs in some patients who later develop MD. The open question in the field is how to distinguish the ALHL without further health consequences from ALHL, which will later transform into definite MD. Here, we attempted to answer this question. We performed retrospective study to identify clinical features associating with MD among the patients diagnosed with ALHL.

The main finding of our study is that tinnitus reported by patients during the first presentation with ALHL might be indicative of developing or probable MD, even in the absence of vertigo. However, the closed question used to determine the presence of tinnitus was not followed by specialized tinnitus diagnostic, such as tinnitus-oriented psychoacoustics and psychometry, which was one of the limitations of this study. Nevertheless, our results imply that ALHL as a sole symptom does not predict MD, at least not within 5 years from the first ALHL episode.

Using pure-tone audiometry, we were unable to identify audiometric characteristics, which could be useful to discriminate future MD patients. The audiograms of MD patients vary. At the onset of MD, patients have typically upward sloping audiogram, indicative of low-frequencies hearing loss. However, in the course of disease, a downward sloping or a flat audiogram (pantonal hearing loss) can develop (20–22). Friberg et al. have studied a cohort of 161 MD patients and demonstrated that 21% of them had pantonal hearing loss, 20% had upward sloping audiogram and 28% had a notched audiogram. Fifteen years later, 74% of patients in this cohort had pantonal hearing loss (23). Thomas et al. studied audiograms of 610 MD patients and demonstrated lack of MD-specific audiogram. The pantonal hearing loss was most commonly represented whereas the upward sloping audiogram was identified only in 12% of the cohort (24). In our present study, we have observed the pantonal hearing loss in 19% of the ALHL cases, whereas the remaining patients had an upward sloping audiogram (data not shown). Hearing loss occurring at the MD onset could still be fluctuating and therefore, the audiometry may not be useful as a diagnostic criterion (25). As the disease progresses, the hearing loss worsens, affecting particularly the frequency of 500 Hz (20), which stabilizes after 5–10 years from the disease onset at a hearing threshold ranging between 50 and 60 dB and speech discrimination of 50–60% as compared to healthy subjects.

A study, where the patients diagnosed with either ALHL, AHHL or with MD were retrospectively analyzed, has determined that 9% of 184 patients with ALHL have developed MD over a course of time (15). Similar conclusions were drawn in other work, where 45 patients with AHLH but without vertigo were followed for 3 years and where 11% of these patients have developed MD (26). In our present study, 24 patients (23%) with AHLH met the diagnostic criteria of MD at presentation and further five patients (4.7%), who presented with ALHL and tinnitus, developed MD over the follow-up time. Interestingly, we observed increased incidence of MD in patients who presented with recurrent ALHL (31.7%), as in the group presenting with ALHL for the first time (16.9%). In the control AHHL group, one patient reported vertigo and was identified as probable MD. Our results corroborate earlier finding of Junicho et al. who demonstrated that 6% of AHHL patients may develop Menière's disease over a course of time (15).

Direct comparison of ALHL and AHHL groups suggested that vertigo alone as well as vertigo with tinnitus–both were found significantly more often in the ALHL group. Furthermore, the recurrence of hearing loss episodes was seen more frequently in ALHL than in AHHL group. Not much is known on this topic in the literature because the ALHL is relatively rare. In addition, diverse classification systems of ALHL used in various countries complicate the matter (27, 28). In addition, the presence of tinnitus in ALHL patients was rarely determined and taken under account as a diagnostic factor. The four-panel charts have demonstrated that tinnitus is not a suitable parameter characterizing ALHL in patients with an acute hearing loss. However, the sensitivity for the comorbid symptom vertigo (alone and in combination with tinnitus) was quite high with 77.5 and 76.5 %. ALHL has therefore a high predictive value for the recurrence of event.

Presence of comorbid conditions is an important parameter in sub-classification of MD. We have screened the patients medical files that were available to us for the presence of such diseases, namely for comorbid migraine and autoimmune diseases. Although no mention of migraine history was found in the obtainable medical information, five ALHL patients had autoimmune diseases (Hashimoto, Sjörgens and type 1 diabetes). Two patients (one with Hashimoto diseases and one with type 1 diabetes) belonged to the group of 24 subjects diagnosed with MD at the time of presentation, thus, confirming the findings of others (19). Further three patients presented with ALHL for the first time but have not given their consent for follow-up. Our finding regarding this aspect of ALHL diagnosis suggest a need for improvement of medical information collection. In fact, we develop ALHL- and MD-specific extended questionnaire, which upon approvement by our local ethics committee will be used in our clinical routine. Some of the questions will specifically deal with autoimmune diseases and migraine symptoms.

The main limitation of our study is its retrospective design and—partly as a consequence of the design–a small number of patients who gave their consent for follow-up. Future, prospective studies should overcame this limitation. The second limitation of our study is the small sample size and a very small group of patients who were followed up after the first episode of ALHL. The first one is a consequence of the ALHL incidence whereas the second results from the fact that many patients came to in our ER from out of town and after initial diagnosis, they were taken over by a local specialist. Designing large multicenter studies could overcome this obstacle. Third limitation of our study is very restricted tinnitus-oriented diagnostics. Including tinnitus-oriented psychoacoustics and psychometric instruments in the future study would add valuable measurable information on the topic.

In addition to vertigo and tinnitus, the ALHL remains an important diagnostic criterion of MD. Other methods used to diagnose MD, which include caloric test, electrocochleography (ECochG), vestibular evoked myogenic potential (VEMP's) and the video Head Impulse Test (vHIT), often produce diagnostic hints, but their sensitivity and specificity demonstrated so far in clinical studies remains limited (29). The new diagnostic method with a use of MRI and Gadolinium contrast media is useful for the imaging of endolymphatic hydrops (30, 31), but the MD symptoms not always correlate with the imaging results (29).

Our findings strongly imply the need for follow-up of patients presenting with ALHL and tinnitus, because of their increased risk to develop MD. To further study the association between the ALHL, occurrence of comorbid symptoms and the positive development of MD, large prospective multicenter and longitudinal studies are essential.

## Author contributions

KS, LV, and AS planned and designed experiments. KS, LV, and JD collected the data. KS and AS analyzed the data. KS and AS wrote the main manuscript. All the authors discussed the data and reviewed the manuscript.

### Conflict of interest statement

The authors declare that the research was conducted in the absence of any commercial or financial relationships that could be construed as a potential conflict of interest.
